# Clinical Manifestation and Multidisciplinary Treatment Approaches for Primary Tracheal Carcinoma in Bangladesh: A Clinical and Therapeutic Review of 13 Patients

**DOI:** 10.1002/cnr2.2135

**Published:** 2024-08-19

**Authors:** Qamruzzaman Chowdhury, Md. Arifur Rahman, Ferdous Ara Begum, Md. Shariful Islam, Murtaza Khair, Zakir Hossain Sarker, Mashud Parvez, A. K. M. Akramul Haque, Ali Hossain

**Affiliations:** ^1^ Department of Oncology Bangladesh Specialized Hospital Limited Dhaka Bangladesh; ^2^ Department of Radiation Oncology National Institute of Cancer Research & Hospital Dhaka Bangladesh; ^3^ Department of Respiratory Medicine Bangladesh Specialized Hospital Limited Dhaka Bangladesh; ^4^ Department of Laboratory Medicine Bangladesh Specialized Hospital Limited Dhaka Bangladesh; ^5^ Department of Thoracic Surgery Bangladesh Specialized Hospital Limited Dhaka Bangladesh

**Keywords:** clinical manifestations, diagnosis and treatment, multidisciplinary treatment, primary tracheal carcinoma, radiotherapy

## Abstract

**Background:**

Primary tracheal carcinoma is an exceptionally rare and life‐threatening disease that presents significant diagnostic and therapeutic challenges. Delayed diagnosis due to misinterpretation of airway obstruction symptoms often leads to poorer prognoses for patients. This study aimed to explore the clinical manifestations and multidisciplinary treatment approaches for primary tracheal carcinoma in Bangladesh, with a focus on recent advancements in diagnosis and treatment.

**Methods:**

A retrospective observational study was conducted at Bangladesh Specialized Hospital Limited, involving patients aged over 30 who were diagnosed with tracheal carcinoma and underwent multidisiplinary treatment from July 2018 to June 2019. Data were collected through patient interviews and medical record reviews. Descriptive and inferential statistical analyses were performed to examine demographic characteristics, histological variations, tumor locations, clinical signs and symptoms, treatment approaches, and outcomes.

**Results:**

The study illuminated varied clinical presentations and the successful application of multidisciplinary approaches among the 13 patients. Invasive squamous cell carcinoma and adenoid cystic carcinoma were the predominant histological subtypes. Symptomatology, including dyspnea, cough, and hemoptysis, highlighted the challenge of early detection. Despite the rarity and intricacies associated with primary tracheal carcinoma, the multidisciplinary strategy yielded generally positive outcomes, as evidenced by a 1‐year survival rate of 92.31% and a 5‐year survival rate of 76.92%. Kaplan–Meier survival curves underscored the superior efficacy of surgical interventions over non‐surgical approaches.

**Conclusion:**

Despite some limitations, this study contributes crucial insights into the nuanced management of primary tracheal carcinoma in the Bangladeshi context. The demonstrated success of the multidisciplinary strategy, especially surgical interventions, accentuates the importance of definitive resection. The lone case of local recurrence emphasizes the necessity for vigilant follow‐up.

## 1 Introduction

Primary tracheal carcinoma is a rare and often deadly disease that poses significant diagnostic and therapeutic challenges. It accounts for less than 0.2% of all malignancies [1], with most tracheal tumors being malignant and endangering the patient's life by obstructing the airway and spreading to other parts of the body. This disease is estimated to occur at a rate of one in a million [1, 2, 3]. Unfortunately, symptoms of airway obstruction are often misdiagnosed as adult‐onset asthma, leading to delayed diagnosis and a poorer prognosis for patients it usually takes to diagnosis several months or even years [4]. Treatment options for primary tracheal carcinoma depend on the stage and extent of the disease. Even though surgical resection is the preferred treatment approach and offers the best chance for long‐term disease control [2, 5], it is not always feasible for patients with advanced or unresectable tumors. In these cases, a combination of therapies, including chemotherapy and radiation, may be necessary to preserve the probability of long‐term disease control. Despite these advanced treatments, the prognosis for patients with primary tracheal carcinoma remains poor and requires a distinct therapeutic approach due to its propensity for submucosal and perineural expansion beyond the tumor's apparent limits [6, 7, 8, 9]. Early detection of primary tracheal tumors is challenging, as they typically present with no specific clinical signs or symptoms. Furthermore, in Bangladesh, there is a lack of specialized centers and expertise in managing primary tracheal carcinoma. Therefore, further research is necessary to improve diagnostic and therapeutic strategies. This study aimed to identify the clinical manifestations and multidisciplinary treatment approaches in treating primary tracheal carcinoma in Bangladesh, with a focus on recent advances in diagnosis and treatment.

## 2 Methods and Materials

### 2.1 Study Design and Patient Selection

We conducted a single‐center hospital‐based retrospective observational study with confirmed cases of primary tracheal carcinoma through a diagnostic procedure. Between July 2018 and June 2019, Bangladesh Specialized Hospital Limited in Dhaka diagnosed all patients aged 30 and above who sought cancer treatment at the hospital's inpatient and outpatient departments of oncology. Primary tracheal carcinoma is relatively rare, and its incidence tends to increase with age. By focusing on patients over 30 years old, we aimed to capture a cohort that is more likely to present with tracheal carcinoma. The diagnoses were confirmed through imaging and histopathological testing, and the patients underwent multidisciplinary treatments after meeting specific inclusion and exclusion criteria. Ethical clearance for the study was obtained from the Institutional Ethical Review Board (IERB) of Bangladesh Specialized Hospital Limited, and written informed consent was obtained from all participating patients.

### 2.2 Histological Grading Classification

Histological grading was conducted according to the WHO grading system, which assesses key features such as nuclear pleomorphism, mitotic rate, and glandular differentiation.

- Grade I (well‐differentiated): Tumors in this grade exhibit minimal nuclear pleomorphism, a low mitotic rate, and well‐formed glands. Adenoid cystic carcinoma was classified as Grade – 1 in this study.
- Grade II (Moderately differentiated): Tumors in this grade show moderate nuclear pleomorphism, moderate mitotic activity, and recognizable glandular structures. Adenocarcinoma with a papillary component was classified as Grade – 2.
- Grade III (poorly differentiated): Tumors in this grade display marked nuclear pleomorphism, a high mitotic rate, and loss of glandular differentiation. Invasive squamous cell carcinoma cases were classified as Grade – 3.

### 2.3 Intervention

#### 2.3.1 Definitive Surgery

“Definitive surgery” refers to a surgical procedure that is performed with the primary intent to completely remove a tumor or lesion. The term “definitive” in this context implies a comprehensive and curative approach to excise the entire tumor, leaving no visible evidence of disease. For the purpose of the study, “definitive surgery” was defined as the radical resection of the affected area along with the removal of portal lymph nodes.

#### 2.3.2 Apple Coring Surgery

The “apple coring” surgical technique involves the removal of a cylindrical or core‐like portion of tissue from a tubular structure, such as the trachea. In the context of tracheal surgery, “apple coring” is a term used to describe a specific type of resection where a segment of the trachea, often resembling the core of an apple, is excised. This technique is employed to address tumors or lesions in the trachea through the following steps.

- Incision and Exposure: An incision is made in the neck or chest, depending on the location of the tracheal tumor. The choice of incision aims to provide the surgeon with the best access to the affected area. After making the incision, the surgeon carefully exposes the trachea, ensuring a clear view of the tumor and the surrounding healthy tissue.
- Identification and Resection: The surgeon identifies the extent of the tumor and performs a circumferential resection of the affected segment of the trachea. This involves removing a cylindrical or sleeve‐shaped portion of the trachea containing the tumor. Preoperative imaging may guide the surgeon in determining the precise location and boundaries of the lesion. This resection aims to achieve complete removal of the diseased tissue.
- Closure or Reconstruction: Following the removal of the tracheal segment, the remaining trachea is often closed or reconstructed. This reconstruction may involve suturing the ends directly or using additional graft material to ensure a secure and patent airway. Closure involves suturing the cut ends of the trachea together.
- Airway Reinforcement: Depending on the extent of the resection, additional measures may be taken to reinforce the airway and prevent complications such as collapse. This could involve the use of stents or other supportive materials.
- Postoperative Care: The patient undergoes postoperative care, including monitoring for complications, managing pain, and ensuring proper healing of the surgical site.

#### 2.3.3 Resection Classification

Resection classification refers to the categorization of surgical procedures based on the extent to which the tumor or affected tissue is removed. We categorized the resection margins following the guidelines of the Royal College of Pathologists [10]. The classification system typically includes three categories: R0, R1, and R2. R0 resection, often termed “complete resection,” indicates that the surgeon successfully removed the entire tumor without any visible or microscopic residual disease, leading to a high likelihood of complete eradication. R1 resection implies that there is microscopic evidence of tumor cells at the surgical margin, suggesting incomplete removal. R2 resection, the least favorable outcome, indicates that macroscopic tumor remnants are left behind due to the inability to remove the entire mass.

#### 2.3.4 Data Collection

A detailed data acquisition strategy was employed, utilizing a meticulously designed pre‐structured Case Record Form (CRF) to ensure the systematic compilation of pertinent information. The data amalgamation process involved a dual methodology—patient interviews and a thorough review of medical records. Patient interviews were conducted to extract essential demographic details such as age and gender, as well as to document symptoms encompassing but not limited to cough, hoarseness, difficulty breathing, and other respiratory‐related manifestations. Simultaneously, pertinent information was extracted from medical records, encompassing clinical features and diagnostic findings derived from various modalities such as computed tomography (CT) scans, magnetic resonance imaging (MRI), bronchoscopy reports, biopsy findings, and histopathological confirmations. The dataset also encapsulates the diverse spectrum of treatment modalities undertaken, ranging from surgical interventions to adjuvant therapies like chemotherapy or radiation. Additionally, outcomes spanning post‐operative recovery, complications, disease recurrence, and long‐term survival were meticulously documented.

#### 2.3.5 Duration of Follow‐Up and Survival Period

The duration of follow‐up for each patient is extended for a maximum of 5 years from the initiation of the multidisciplinary treatment. This follow‐up period was chosen to allow for a comprehensive evaluation of treatment outcomes, including the detection of any potential late recurrences or patient expired. Follow‐up appointments were scheduled at regular intervals, and imaging studies were conducted periodically to monitor the patient's health status and detect any signs of disease recurrence. Additionally, patient records, including medical history, imaging reports, and pathology findings, were meticulously reviewed to ensure the accuracy and completeness of the follow‐up data. The comprehensive approach to follow‐up aimed to capture both short‐term and long‐term treatment outcomes, contributing to a thorough assessment of the multidisciplinary treatment's efficacy.

#### 2.3.6 Determining Local Recurrence and Treatment Outcomes

Local recurrence was assessed through a combination of clinical evaluation, imaging studies, and, when feasible, histopathological confirmation. The clinical evaluation involved regular follow‐up appointments wherein patients were examined for signs and symptoms indicative of local recurrence. Additionally, imaging modalities, such as computed tomography (CT) scans, were employed to detect any anatomical or structural changes suggestive of recurrent disease. In cases where suspicious findings were noted, histopathological confirmation through biopsy or other appropriate diagnostic procedures was pursued. We defined treatment outcomes as patients classified as “alive” who have survived beyond a specified follow‐up period post‐treatment, indicating a positive response to the multidisciplinary therapeutic approach. On the other hand, patients classified as “expired” have unfortunately succumbed to the disease during the post‐treatment follow‐up period.

### 2.4 Data Analysis

The gathered data underwent meticulous review and subsequent cleaning procedures to ensure accuracy and consistency. The process involved the identification and removal of errors, inconsistencies, and missing values present in the dataset. Statistical analysis was conducted to derive meaningful insights from the detailed dataset collected during the study. The analysis aimed to explore the outcomes of multidisciplinary treatments for primary tracheal carcinoma, considering various factors such as patient age, histological variations, and treatment approaches.

Descriptive statistics were employed to characterize the demographic profile of the study population. The age, gender, and histological variations were summarized using means, and standard deviations, when appropriate. The distribution of histological types among different age groups was assessed to discern potential age‐related patterns. The primary focus of the analysis was on treatment outcomes, with a special emphasis on survival rates. Survival analysis was conducted to evaluate the overall survival of patients over the follow‐up period. Kaplan–Meier survival curves were generated to illustrate the overall survival of the 13 patients. All statistical analyses were conducted using MS Excel and STATA (version 13).

## 3 Results

A total of 13 patients diagnosed with primary tracheal carcinoma were included in the study. The demographic characteristics of these patients are summarized in Table 1. The age of the patients ranged between 32 and 63 years, with a mean (±SD) age of 49.15 ± 10.50 years. Among the age groups, the highest proportion of patients was observed in the age groups of 41–60 years and above 60 years, each accounting for 30.78% of the total, followed by the age groups of 31–40 years and 41–50 years, representing 23.08% and 15.38% of the patients, respectively. Among the 13 patients, 6 (46%) were male and 7 (54%) were female (Table 1).

**TABLE 1:** Distribution of demographic information of the patients with primary tracheal carcinoma (*n* = 13).

Characteristics	*n*	%
Age in years		
31–40	03	23.08
41–50	04	30.78
51–60	02	15.38
60+	04	30.78
Sex		
Male	06	46
Female	07	54

Among the histological variations observed in the primary tracheal carcinomas, the most common subtypes were invasive squamous cell carcinoma and adenoid cystic carcinoma, each of the subtypes accounting for 30.78% (*n* = 4) of the total cases. Regarding the invasive squamous cell carcinoma, the two cases were classified as Grade – 3, while the two cases of adenoid cystic carcinoma were classified as Grade – 1. Adenoid cystic carcinoma was also equally divided between Grade – 1 and cases where the grade was not specified due to pathological reporting variation. Likewise, there were 2 cases (15.38%) of adenocarcinoma with a papillary component, both of which were classified as grade – 1. Carcinoid tumor, mucoepidermoid carcinoma, and neuroendocrine carcinoma each represented 7.69% (*n* = 1) of the total cases. The mucoepidermoid carcinoma was also Grade – 1. The remaining carcinoid tumor and neuroendocrine carcinoma did not have specified grades. The majority of the observed tracheal carcinomas were located in the upper trachea, accounting for 53.85% of the cases. The lower trachea was the second most common location, representing 38.46% of the cases. Only one patient had a tumor located in the mid‐trachea, making up 7.69% of the cases (Table 2).

**TABLE 2:** Age sex, histological variations, grade, and tumor position of primary tracheal carcinoma for each of the 13 patients.

Patient no.	Age	Sex	Histological variations	Grade	Tumor position
1	37	Male	Invasive squamous cell carcinoma	Grade – 3	Upper
2	32	Female	Adenoid cystic carcinoma, PNI, and LVSI absent		Lower
3	51	Female	Adenoid cystic carcinoma, PNI, and LVSI absent		Lower
4	46	Female	Adenoid cystic carcinoma	Grade – 1	Lower
5	42	Female	Adenoid cystic carcinoma	Grade – 1	Upper
6	63	Female	Suggestive of carcinoid tumor		Upper
7	63	Female	Invasive squamous cell carcinoma	Grade – 3	Upper
8	34	Male	Adenocarcinoma with papillary component	Grade – 1	Upper
9	60	Male	Adenocarcinoma with papillary component	Grade – 1	Upper
10	48	Male	Invasive squamous cell carcinoma, LVSI absent	Grade – 1	Lower
11	46	Male	Mucoepidermoid carcinoma	Grade – 1	Lower
12	56	Male	Invasive squamous cell carcinoma	Grade – 1	Middle
13	61	Female	Neuroendocrine carcinoma		Upper

Abbreviations: LVSI, lymph‐vascular space invasion; PNI, perineural invasion.

**FIGURE 1:**
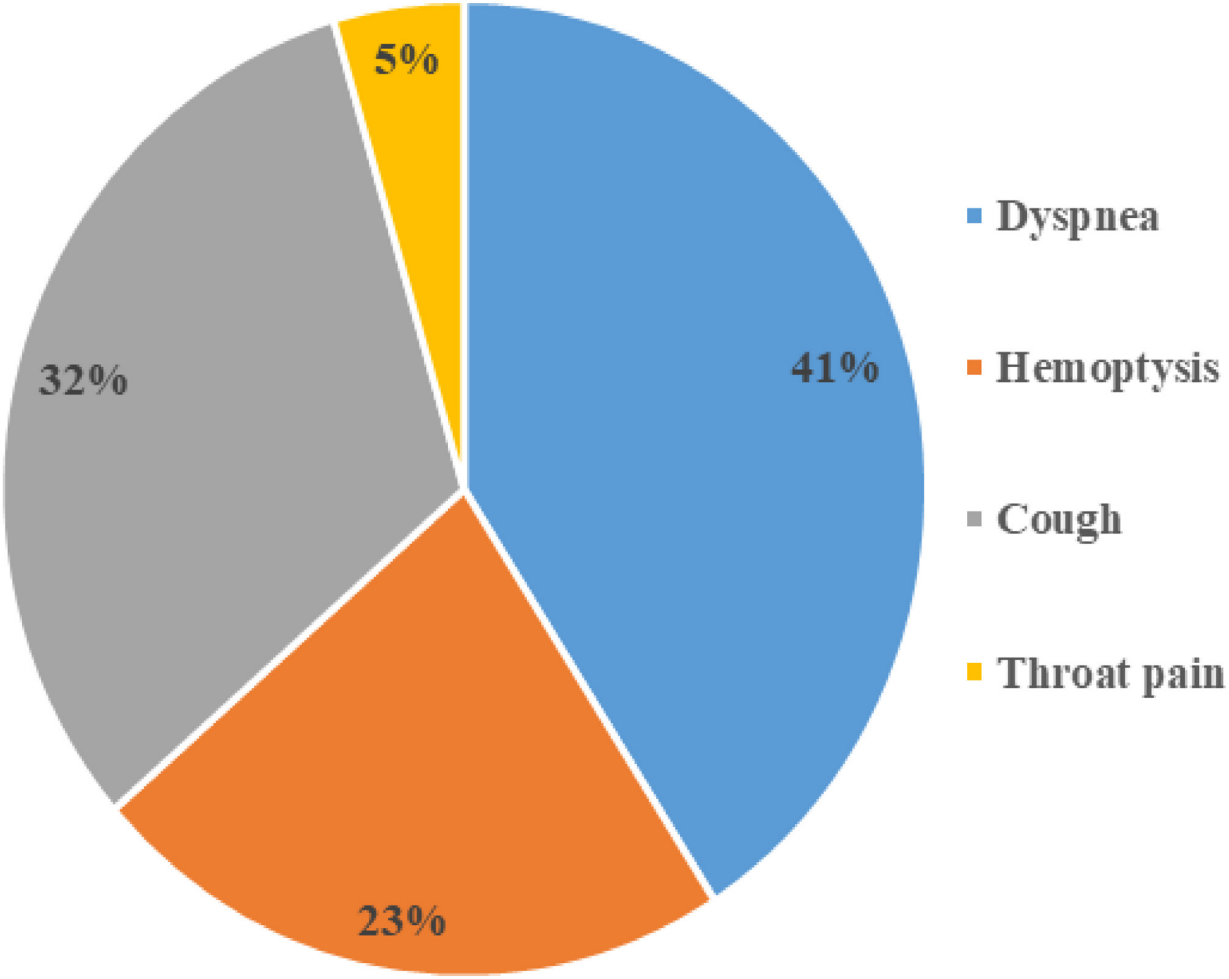
Signs and symptoms.

Among the reported symptoms regardless of the tumor locations, dyspnea (difficulty in breathing) was the most prevalent, observed in 9 out of the 13 patients (69.23%). Cough was reported by 7 patients, accounting for a prevalence of 53.84%. Hemoptysis (coughing up blood) was observed in 5 patients, representing a prevalence of 38.46%. Throat pain was reported by only one patient, accounting for a prevalence of 7.69% of the cases. However, no cases of Dysphagia were reported among patients with tracheal carcinoma (Figure 1).

### 3.1 Multidisciplinary Treatment Approach

Thirteen patients diagnosed with primary tracheal carcinoma underwent a comprehensive range of multidisciplinary treatments, including surgical interventions such as Apple Coring Surgery and Definitive Surgery, as well as non‐surgical treatments including Recanalization + Cryo + APC, Cryo, and Snare Diathermy. The surgical interventions were carried out through either a neck incision or a thoracotomy. Among the 13 patients, seven individuals (53.85%) received surgical treatment, which was the most commonly used treatment approach, while some patients were treated with nonsurgical approaches, after treatment with adjuvant external beam radiotherapy (EBRT), with varying doses and fractions tailored to individual cases. Out of the surgical approaches, three (23.08%) received definitive surgery, while four (30.77%) underwent apple coring surgery. while out of the 13 patients, five individuals underwent recanalization in combination with cryoablation (Cryo) and argon plasma coagulation (APC), or cryoablation (Cryo) followed by adjuvant external beam radiotherapy (EBRT). Out of these five patients, three (23.08%) underwent recanalization along with cryoablation (Cryo) and argon plasma coagulation (APC), two (15.38%) received cryoablation alone, and an additional patient (7.69%) received chemotherapy followed by snare diathermy. The delivery of EBRT utilized conformal 3D CRT, with doses ranging from 50 to 66 Gy based on the prescribed settings (Table 3).

**TABLE 3:** Receiving multiple treatment approaches, survival time and patient outcome for each of the 13 patients.

Patient no.	Multiple treatment approaches	Accessed method	Adjuvant radiation (dose/fraction)	Survival time (month)	Patient outcome	Local recurrence
1	Apple coring surgery	Neck incision	EBRT (60 Gy/30#)	60	Expired	No
2	Recanalization + Cryo + APC		EBRT (66 Gy/33#)	60	Alive	No
3	Definitive surgery	Thoracotomy	EBRT (54 Gy/27#)	60	Alive	No
4	Definitive surgery	Thoracotomy	EBRT (54 Gy/27#)	60	Alive	No
5	Apple coring surgery	Neck incision	EBRT (60 Gy/30#)	60	Alive	No
6	Recanalization + Cryo + APC		EBRT (60 Gy/30#)	33	Expired	No
7	Apple coring surgery	Neck incision	EBRT (60 Gy/30#)	50	Alive	No
8	Recanalization + Cryo + APC		EBRT (60 Gy/30#)	51	Alive	No
9	Definitive surgery	Neck incision	EBRT (56 Gy/28#)	60	Alive	No
10	Cryo		EBRT (54 Gy/27#)	09	Expired	Yes
11	Cryo		EBRT (55.8 Gy/31#)	31	Alive	No
12	Apple coring surgery	Neck incision	EBRT (50 Gy/25#)	27	Alive	No
13	Snare diathermy		Chemotherapy	07	Alive	No

Abbreviations: APC, argon plasma coagulation; Cryo, cryoablation; EBRT, external beam radiotherapy.

Among the patients, seven individuals (46.15%) underwent surgery, the most frequently used treatment approach after treatment with adjuvant external beam radiotherapy (EBRT). Regarding the surgical approaches, three (23.08%) received definitive surgery, while four (30.77%) underwent apple coring surgery. On the other hand, among the 13 patients, five individuals received recanalization in combination with cryoablation (Cryo) and argon plasma coagulation (APC), or cryoablation (Cryo) followed by adjuvant external beam radiotherapy (EBRT). Out of these five patients, three (23.08%) underwent recanalization along with cryoablation (Cryo) and argon plasma coagulation (APC), two (15.38%) received cryoablation alone, and an additional patient (7.69%) received chemotherapy followed by Snare Diathermy. The delivery of EBRT utilized conformal 3D CRT, with doses ranging from 54 to 66 Gy based on the prescribed settings (Table 3).

### 3.2 Detailed Outcomes

Thirteen patients diagnosed with primary tracheal carcinoma underwent a comprehensive multidisciplinary treatment approach, resulting in varying survival times ranging from 9 to 60 months. The outcomes reflected a spectrum of experiences, including instances of expiration and continued survival, highlighting the diverse nature of patient responses. Notably, the majority of patients exhibited positive responses to the treatment regimen. Remarkably, only one patient (Patient 10) (7.69%) encountered local recurrence, presenting a unique clinical perspective. This patient underwent close monitoring over a 60‐month follow‐up period; however, unfortunately, succumbed to the condition within 9 months. Additionally, patients 1 and 6 also experienced expiration at 60 and 33 months, respectively. Among the entire cohort, 10 patients (76.92%) persisted in their battle against the disease, while three patients (23.08%) eventually succumbed, as depicted.

### 3.3 Surgical Procedures and Resection Outcomes

We performed various surgical procedures on a group of 13 patients to ensure optimal resection outcomes. Patients 3, 4, and 9 underwent definitive surgery through thoracotomy for the first two and a neck incision for the last one, respectively, resulting in the complete removal of the tumor with negative margins. While patients 1, 5, 7, and 12 underwent apple coring surgery through a neck incision. Our study did not observe any instances of R1 resection, and we made all efforts to achieve R0 status. Unfortunately, Patient 1 underwent apple coring surgery with access through a neck incision and experienced R2 resection, indicating the presence of macroscopic residual tumor tissue.

### 3.4 Survival Rate

Our analysis showed that the patients who underwent surgical or non‐surgical intervention had no deaths within 30 days of surgery, indicating a high level of safety. Moreover, we observed a 92.31% survival rate at 1 year, which highlights the effectiveness of our multidisciplinary approach during the initial phase of treatment. Equally compelling, the 5‐year survival rate was found to be 76.92%, demonstrating the long‐lasting benefits of our approach. We calculated the survival rates using specific formulas and based them on patients who successfully completed the follow‐up period. Our findings emphasize the clinical relevance and sustained positive impact of our multidisciplinary treatment strategy on primary tracheal carcinoma.

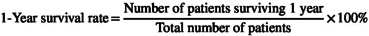




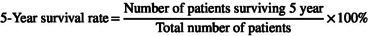




### 3.5 Kaplan–Meier Survival Estimates

**FIGURE 2:**
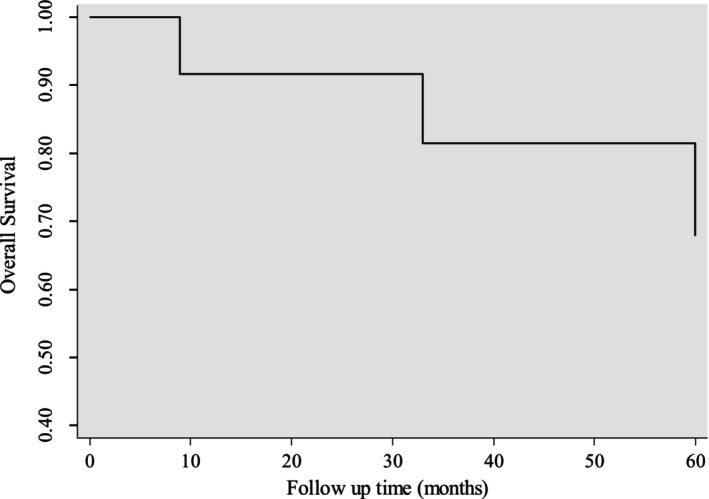
Kaplan–Meier survival curve for multiple treatment approach.

Plotting the survival curve by plotting the cumulative proportion of survivors against the survival times is shown in Figure 2.

**FIGURE 3:**
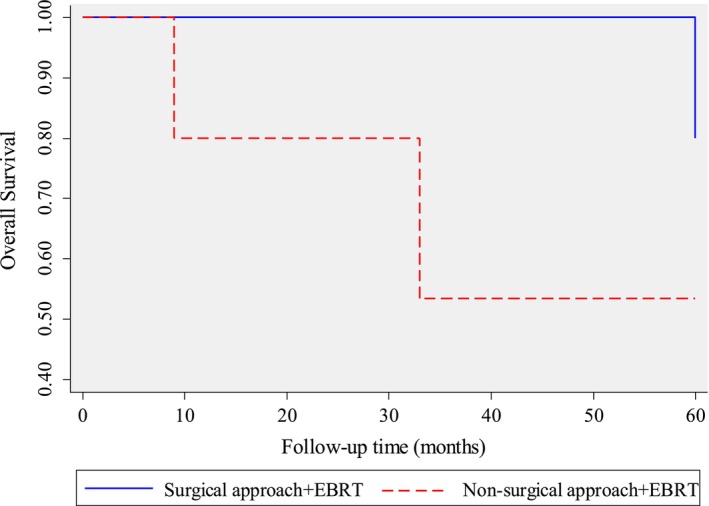
Kaplan–Meier survival curve for the two treatment approaches.

Based on Figure 3, it seems that the patients who were treated surgically had a greater chance of survival compared to those who were treated non‐surgically. However, the graph cannot be relied upon to determine the median survival time, which is the time at which there is a 50% chance of survival. As the survival curve did not cross 50%, the median survival is undefined and greater than the last time point.

## 4 Discussion

Tracheal carcinoma is a rare malignancy that poses significant challenges in diagnosis and treatment. In this study, we investigated the demographic characteristics, histological variations, tumor locations, clinical signs and symptoms, treatment approaches, and outcomes of patients with primary tracheal carcinoma.

The demographic characteristics of the 13 patients included in the study revealed a wide age range, with a mean age of 49.15 ± 10.50 years, indicating that the Bangladeshi population is extremely prone to primary tracheal cancer at the age of 49. Other studies reported higher mean age (>60 years) compared to the current study [11]. Due to the very small sample, our study group may not be fully representative of the broader population affected by primary tracheal carcinoma. The highest proportion of our study patients was observed in the age groups of 41–60 years and above 60 years, each accounting for 30.78% of the total cases. This distribution suggests that tracheal carcinoma can affect individuals across various age group. In terms of gender, the study population consisted of 46% males and 54% females, indicating a relatively balanced representation. The previous study related to Primary tracheal malignant neoplasms also found among 74 cases, 55.4% were male and 44.6% were female [12].

Histologically, the most common subtypes observed in primary tracheal carcinoma were invasive squamous cell carcinoma and adenoid cystic carcinoma, each accounting for 30.78% of the cases. Based on these findings, it can be inferred that Bangladeshi adults are more susceptible to both conditions compared to other histological variations of carcinoma. This higher incidence may be attributed to factors such as dietary habits, smoking, lifestyle choices, or other related issues prevalent in the population. These findings are consistent with the known histological variations observed in tracheal carcinoma [13]. The grading of the tumors varied, with the invasive squamous cell carcinoma cases classified as Grade – 3, while the adenoid cystic carcinoma cases were Grade – 1. Adenocarcinoma with a papillary component, carcinoid tumor, mucoepidermoid carcinoma, and neuroendocrine carcinoma was also identified, albeit less frequently.

The distribution of tumor locations revealed that the majority of tracheal carcinomas were located in the upper trachea (53.85%), followed by the lower trachea (38.46%), and with only one case observed in the mid trachea (7.69%). This distribution highlights the predominance of upper tracheal involvement in the cases of tracheal carcinoma examined in this study, followed by the lower trachea. These findings provide valuable insights into the anatomical distribution of tracheal carcinomas and may have implications for diagnosis, treatment planning, and clinical management strategies.

The analysis of symptoms revealed dyspnea as the predominant clinical manifestation, aligning with the literature [14]. Dyspnea, hemoptysis, and cough were the most commonly reported symptoms. Dyspnea was observed in varying frequencies among different tumor locations, with 100% prevalence in patients with lower tracheal carcinoma. Hemoptysis was also reported, but its occurrence varied among different tumor locations. The absence of dysphagia suggests the unique clinical profile of primary tracheal carcinoma, distinguishing it from malignancies affecting adjacent structures. Throat pain was reported by one patient with upper tracheal carcinoma. Overall, the observed signs and symptoms align with the typical clinical presentation of tracheal carcinoma.

Among the reported symptoms, dyspnea was the most prevalent, observed in 9 out of the 13 patients (69.23%). This finding suggests that dyspnea is a common symptom experienced by individuals with tracheal carcinoma, regardless of the tumor location. Cough was the second most frequently reported symptom, observed in 7 patients (53.84%). Hemoptysis (coughing up blood) was reported by 5 patients (38.46%), while throat pain was reported by only one patient (7.69%) (Figure 3). Dyspnea was observed across all tumor locations, albeit with varying frequencies. Among patients with upper tracheal carcinoma, 3 out of 7 patients (42.85%) experienced dyspnea. In the group with lower tracheal carcinoma, all 5 patients (100%) reported dyspnea. The single patient with mid‐tracheal carcinoma also reported dyspnea. These findings suggest that dyspnea may be more prevalent in patients with lower tracheal carcinoma which are consistent with previous studies that highlighted the variability of symptoms based on tumor location [15]. Cough, another common clinical sign, was reported in both the upper and lower tracheal carcinoma groups. Among patients with upper tracheal carcinoma, 4 out of 7 patients (57.14%) had a cough, while 3 out of 5 patients (60%) with lower tracheal carcinoma presented this symptom. However, no patients with mid‐trachal carcinoma reported coughing. This suggests that the presence of a cough may vary depending on the specific tumor location. Dysphagia and throat pain were less common symptoms in this study. The lack of significant association between tumor location and symptoms suggests that other factors may influence the presentation of signs and symptoms in tracheal carcinoma cases.

An elaborate multidisciplinary treatment strategy was employed, encompassing both surgical and non‐surgical modalities. Surgical interventions, particularly definitive surgery and apple coring surgery, were predominantly performed, followed by a subset of patients who underwent recanalization coupled with cryoablation and argon plasma coagulation, and external beam radiotherapy (EBRT). Chemotherapy combined with Snare Diathermy was also used in one patient. These findings highlight the landscape of therapeutic options employed for primary tracheal carcinoma, with variations in surgical procedures, endobronchial interventions, radiation techniques, and the incorporation of chemotherapy in some cases. Similar treatment approaches and strategies were recommended for the treatment of tracheal carcinoma patients in some other studies [16, 17, 18].

The surgical outcomes revealed successful resections with negative margins, except for one instance of R2 resection following apple coring surgery. This highlights the challenges in achieving complete resection in certain cases, necessitating ongoing refinement of surgical techniques. The survival analysis demonstrated favorable outcomes, with a 92.31% 1‐year survival rate and a robust 76.92% 5‐year survival rate. These rates are aligned with a study conducted in the Netherlands, which demonstrated surgical outcomes of adenoid cystic carcinoma (ACC) for 1‐year and 5‐year survival rates 95% and 61% respectively [2]. These findings suggest the efficacy of the multidisciplinary approach, emphasizing its potential for long‐term benefits. The Kaplan–Meier survival curve further illustrated the divergence in survival outcomes between surgical and non‐surgical interventions, supporting the notion that surgical approaches may confer a survival advantage.

## 5 Conclusion

This study offers significant insights into primary tracheal carcinoma, encompassing demographics, varied histological subtypes, and treatment outcomes. The predominant use of surgical interventions, notably Apple Coring Surgery and Definitive Surgery, coupled with favorable 1‐year (92.31%) and 5‐year (76.92%) survival rates, emphasizes the effectiveness of our multidisciplinary approach. While surgical methods demonstrated superior survival trends, detailed analyses revealed successful resections with the absence of R1 resections. However, the occurrence of R2 resection in one case highlights the complexities of achieving complete resection. Our findings, despite limitations in sample size, contribute valuable clinical knowledge to this rare malignancy, emphasizing the importance of ongoing research and collaborative efforts for refining treatment strategies and enhancing patient outcomes.

### 5.1 Limitation

Despite the valuable insights gained from our study on primary tracheal carcinoma, certain limitations should be acknowledged. The relatively small sample size of 13 patients may affect the generalizability of our findings, warranting cautious interpretation. Additionally, the retrospective nature of the study poses inherent limitations, including potential biases and incomplete data. A larger sample size would provide more robust and reliable results, allowing for a better understanding of the characteristics and outcomes of tracheal carcinoma.

## Author Contributions

**Qamruzzaman Chowdhury** and **Md. Arifur Rahman:** conceptualization, methodology, writing – reviewing and editing. **Md. Arifur Rahman:** writing – original draft preparation, data curation. **Murtaza Khair, Zakir Hossain Sarker, Mashud Parvez, A. K. M. Akramul Haque,** and **Ali Hossain:** visualization, investigation, diagnosis, surgical management. **Ferdous Ara Begum:** medical management, follow up. **Qamruzzaman Chowdhury, Md. Arifur Rahman,** and **Md. Shariful Islam:** radiotherapy, follow up and treatment planning.

## Conflicts of Interest

The authors declare no conflicts of interest.

## Data Availability

Data sharing is not applicable to this article as no new data were created or analyzed in this study.
